# Integrated analysis of single-cell and bulk RNA sequencing data reveals prognostic characteristics of lysosome-dependent cell death-related genes in osteosarcoma

**DOI:** 10.1186/s12864-024-10283-5

**Published:** 2024-04-17

**Authors:** Yueshu Wu, Jun Yang, Gang Xu, Xiaolin Chen, Xiaochen Qu

**Affiliations:** 1 Department of Orthopaedics, First Affiliated Hospital of Dalian Medical University, Dalian, PR China; 2 Key Laboratory of Molecular Mechanism for Repair and Remodeling of Orthopaedic Diseases, Liaoning province, 116011 Dalian, Liaoning, PR China; 3 Department of Orthopedic Surgery, The Second Affiliated Hospital of Chongqing Medical University, No. 76, Linjiang Road, Yuzhong District, 400010 Chongqing, China

**Keywords:** Osteosarcoma, Lysosome-dependent cell death, Prognosis, Immune, Biomarkers

## Abstract

**Background:**

Tumor cells exhibit a heightened susceptibility to lysosomal-dependent cell death (LCD) compared to normal cells. However, the role of LCD-related genes (LCD-RGs) in Osteosarcoma (OS) remains unelucidated. This study aimed to elucidate the role of LCD-RGs and their mechanisms in OS using several existing OS related datasets, including TCGA-OS, GSE16088, GSE14359, GSE21257 and GSE162454.

**Results:**

Analysis identified a total of 8,629 DEGs1, 2,777 DEGs2 and 21 intersection genes. Importantly, two biomarkers (ATP6V0D1 and HDAC6) linked to OS prognosis were identified to establish the prognostic model. Significant differences in risk scores for OS survival were observed between high and low-risk cohorts. Additionally, scores of dendritic cells (DC), immature DCs and γδT cells differed significantly between the two risk cohorts. Cell annotations from GSE162454 encompassed eight types (myeloid cells, osteoblastic OS cells and plasma cells). ATP6V0D1 was found to be significantly over-expressed in myeloid cells and osteoclasts, while HDAC6 was under-expressed across all cell types. Moreover, single-cell trajectory mapping revealed that myeloid cells and osteoclasts differentiated first, underscoring their pivotal role in patients with OS. Furthermore, ATP6V0D1 expression progressively decreased with time.

**Conclusions:**

A new prognostic model for OS, associated with LCD-RGs, was developed and validated, offering a fresh perspective for exploring the association between LCD and OS.

**Supplementary Information:**

The online version contains supplementary material available at 10.1186/s12864-024-10283-5.

## Introduction

Osteosarcoma (OS) represents a highly aggressive malignant bone tumour, predominantly affecting children under nine years old, with an incidence of 2.3 new cases per million in the past decade [1]. Commonly located in the long bones, particularly around the knee, OS manifests with symptoms such as severe pain, bone swelling and, in some cases, pathologic fractures [2]. Approximately 25% of patients are diagnosed with metastatic disease, with the lung being the most frequent site of metastasis [3]. Despite notable advancements in treatment, including the introduction of neoadjuvant and adjuvant chemotherapy in the 1970s, progress in disease-free recurrence and overall survival has plateaued since the 1980s [4]. Moreover, patients with metastatic disease face a grim prognosis, with a 5-year overall survival rate ranging from 10–30% [5]. The turn of the 21st century witnessed significant advancements in the understanding of OS biology, owing to the widespread availability of technologies for comprehensive molecular profiling and well-annotated tissue banks [6]. Consequently, there is a growing need to identify novel prognostic biomarkers that could enhance the survival outcomes of patients with OS and unveil novel therapeutic avenues.

Lysosomes, single-membrane cellular organelles, play crucial roles in macromolecular degradation, plasma membrane repair, antigen presentation, cell surface receptor recycling and apoptotic signalling [7]. The integrity of lysosomal membranes is pivotal for cellular fate as an alteration in membrane permeability leads to the release of various lysosomal enzymes, mainly cathepsins, into the cytoplasm, leading to the degradation of crucial cellular components and/or activation of apoptotic pathways, which is defined as lysosomal-dependent cell death (LCD) and characterized by the rupture of the lysosome [8, 9]. Studies report that cancer cells with lysosomes exhibiting weaker membrane stability than normal cells are more susceptible to lysosomal membrane permeabilization (LMP) and consequent LCD [10, 11]. For instance, Jiang et al. demonstrated that the accumulation of lysosomes, which are subject to increased levels of LMP, facilitates the apoptosis of cells in acute myeloid leukaemia [12]. However, the exact role of LCD in the progression of OS remains unclear. Targeting lysosomes to induce LCD presents a promising strategy for cancer treatment [13], underscoring the urgency to explore LCD-related genes (LCD-RGs) and associated molecular mechanisms in OS.

Herein, leveraging OS-related data from public databases, we employed bioinformatics methodologies to identify prognostic biomarkers associated with LCD in patients with OS. Subsequently, we developed a novel prognostic model to elucidate the biological pathways underlying these prognostic genes and their relationship with clinical characteristics, immune microenvironment and drug sensitivity. Furthermore, we delved into the functional roles of these biomarkers through single-cell analysis, aiming to unveil novel immunotherapy and targeted therapy strategies, thereby offering a new perspective for improving the prognosis of patients with OS.

## Materials and methods

### Data source

**Fig. 1:**
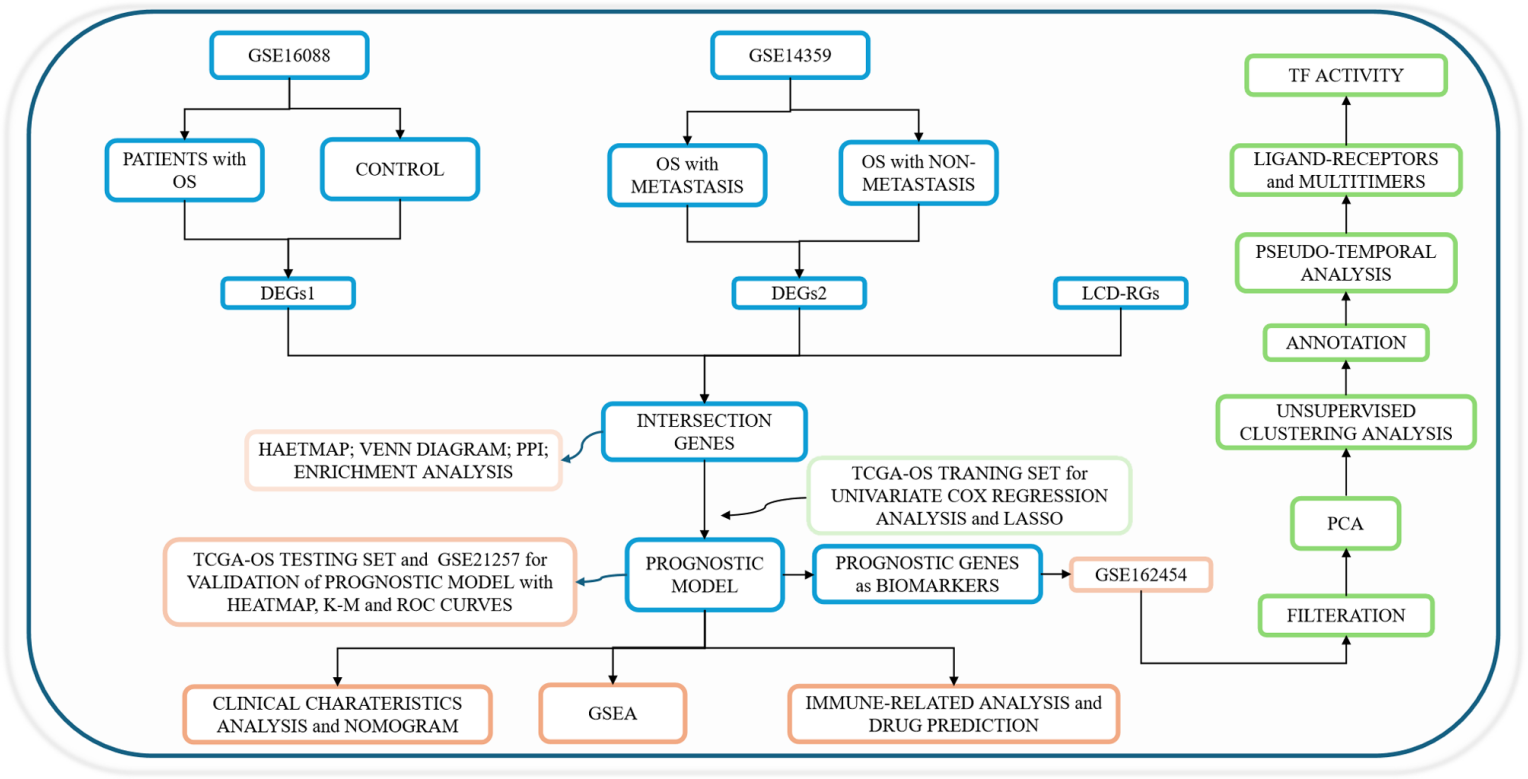
The flowchart for this study. GSE, gene expression omnibus series; DEGs, differentially expressed genes; PPI, protein-protein interaction; LCD-RGs, lysosomal-dependent cell death-related genes; LASSO, least absolute shrinkage and selection operator; TCGA-OS, The Cancer Genome Atlas-osteosarcoma; K-M, Kaplan-Meier; ROC, receiver operating characteristic; GSEA, gene set enrichment analysis; TF, transcription factor

OS dataset was obtained from The Cancer Genome Atlas (TCGA) database (https://portal.gdc.cancer.gov/), comprising 84 patient samples with overall survival. These were divided into training and testing sets in a 5: 5 ratio, with 42 patients each. Additionally, datasets GSE16088 (GPL96), GSE14359 (GPL96), GSE21257 (GPL10295) and GSE162454 (GPL24676) were retrieved from the Gene Expression Omnibus (GEO) database (https://www.ncbi.nlm.nih.gov/geo/), with sample types labelled as tumor. The GSE16088 contained 14 OS samples and six control samples, and GSE14359 included eight lung metastasis and 10 non-metastasis patients with OS. The GSE21257 dataset provided 53 patient samples for external validation, while the single-cell dataset GSE162454 included 6 OS tumour tissues. A total of 220 LCD-RGs were sourced from previously published literature [14]. The specific flowchart of the study was shown in Fig. 1.

### Differentially expressed genes (DEGs) screening and gene enrichment analysis

Differential expression analysis identified DEGs1 between OS and control cohorts in GSE16088 and DEGs2 between metastasis and non-metastasis patients with OS in GSE14359, utilising the limma package (v 3.48.3) [15], with a criteria of adj.*P* < 0.05 and|log2FC| ≥ 0.5. Intersection genes were determined by overlapping DEGs1, DEGs2 and LCD-RGs. A protein-protein interaction (PPI) network was constructed using the STRING website (https://string-db.org) based on these intersection genes. Furthermore, Gene Ontology (GO), encompassing Cellular Components (CC), Molecular Functions (MF) and Biological Process (BP), along with Kyoto Encyclopedia of Genes and Genomes (KEGG) analysis, were performed using the cluster Profilerpackage (v 4.0.2) [16] to elucidate the functional roles of intersection genes.

### Construction and validation of prognostic model

Using the intersection genes, univariate Cox regression analysis (survival package, v 3.2–13) [17] and least absolute shrinkage and selection operator (LASSO) regression analysis (glmnet package, v 4.1-3) [18] were utilized to select prognostic genes as biomarkers in the training set. Risk scores were computed using the formula.

$$ risk\,score=\sum _{i=1}^{n}\left(coefi\text{*}Xi\right){'}$$

where Coef and X represent coefficients and gene expressions, respectively. A prognostic model was developed and evaluated using the proportional hazards (PH) assumption test. Samples from the training set were stratified into high- and low-risk cohorts based on the median risk score, and risk curves and biomarker expression heatmaps were generated using the ggplot2 package (v 3.3.5) [19]. Additionally, Kaplan-Meier (K-M) survival curves were plotted for both cohorts of the training set using the survminer package (v 0.4.9). To further ensure the validity of our risk model, receiver operating characteristic (ROC) curves were constructed for 1, 3 and 5 years, and area under the curves (AUC) values were computed using survivalROC (v 1.0.3) [20]. Validation of the prognostic model was performed using the testing set and GSE21257. Moreover, biomarkers expression in tumour and normal cohorts of GSE16088 and in metastasis and non-metastasis patients with OS of GSE14359 was analysed and compared using box plots.

### Correlation analysis of clinical characteristics and construction of nomogram

The distribution of risk scores among five clinical characteristics (status, age, gender, race and metastatic) was assessed using the Wilcoxon test. Subsequently, univariate Cox regression analysis was conducted for risk score and the five clinical characteristics, with factors having *P* < 0.05 included in multivariate Cox regression analysis to identify independent prognostic factors. A nomogram was constructed based on these factors, and survival rates at 1, 3 and 5 years were predicted. The predictive performance of the nomogram was evaluated using calibration and ROC curves.

### Enrichment analysis of risk cohorts

Gene set enrichment analysis (GSEA) was performed based on log2FC, with threshold settings of|NES| > 1, NOMP < 0.05 and q < 0.25 for all genes in risk cohorts. Enrichment of GO terms (background gene set: c5.go.v7.4.entrez.gmt) and KEGG pathways (background gene set: c2.cp.kegg.v7.4.entrez.gmt) associated with genes in risk cohorts were conducted using the clusterProfiler and org.Hs.eg.db packages (v 3.13.0).

### Immune-related analysis and drug prediction

The single sample GSEA (ssGSEA) algorithm was employed to determine the percentage of 24 types of infiltrating immune cells. Differences in the percentage of infiltrating immune cells between two cohorts were assessed using the Wilcoxon test. Spearman correlation analysis was utilised to examine the relationship between risk scores and significantly different immune cell populations. Additionally, the expression level of immune checkpoints in risk cohorts and their correlation with risk scores and differentially expressed immune checkpoints were investigated using Wilcoxon and Spearman analyses, respectively. The half maximal inhibitory concentration (IC50) values of 198 anticancer drugs were computed and compared based on the Genomics of Drug Sensitivity in Cancer (GDSC) database (https://www.cancerrxgene.org/) through oncoPredict (v 0.2).

### Single-cell analysis

Initially, single-cell data were evaluated, and low-quality cells were filtered out using the Seurat package (v 4.1.0) [15] with parameters set at min.cells = 3 and min.features = 200. The vst method of FindVariableFeatures function was applied to identify highly variable genes for downstream analysis. Subsequently, principal component analysis (PCA) downscaling was conducted to select principal components (PCs) after calibration by harmony (v 0.1.1) [21]. Unsupervised clustering analysis was then performed on the filtered cells using the FindNeighbors and FindClusters functions, and the results were visualised using t-SNE. Clusters were annotated based on cell annotations combined with marker genes from the CellMark database. Next, the expression of biomarkers in different cell types was analysed and illustrated using UMAP and violin plots. Finally, pseudo-temporal analysis was conducted using the Monocle 2 algorithm in Monocle (v 2.24.1) [22] to project different types of core cells onto a root and construct single-cell trajectory maps of biomarkers. Based on the CellPhone DB database, the number of interacting ligand-receptors and multimers between various cell subtypes were computed and filtered to perform cellular communication analysis of single-cell data with a screening threshold of *P* ≤ 0.05, log2mean (Molecule 1 and 2) ≥ 0.1. Additionally, transcription factor (TF) activity in different cell types was calculated using Dorothea (v 1.7.2) [23].

### Statistical analysis

All analyses were conducted using the R programming language, with statistical significance set at *P* < 0.05.

## Results

### Identification and function analysis of DEGs

**Fig. 2:**
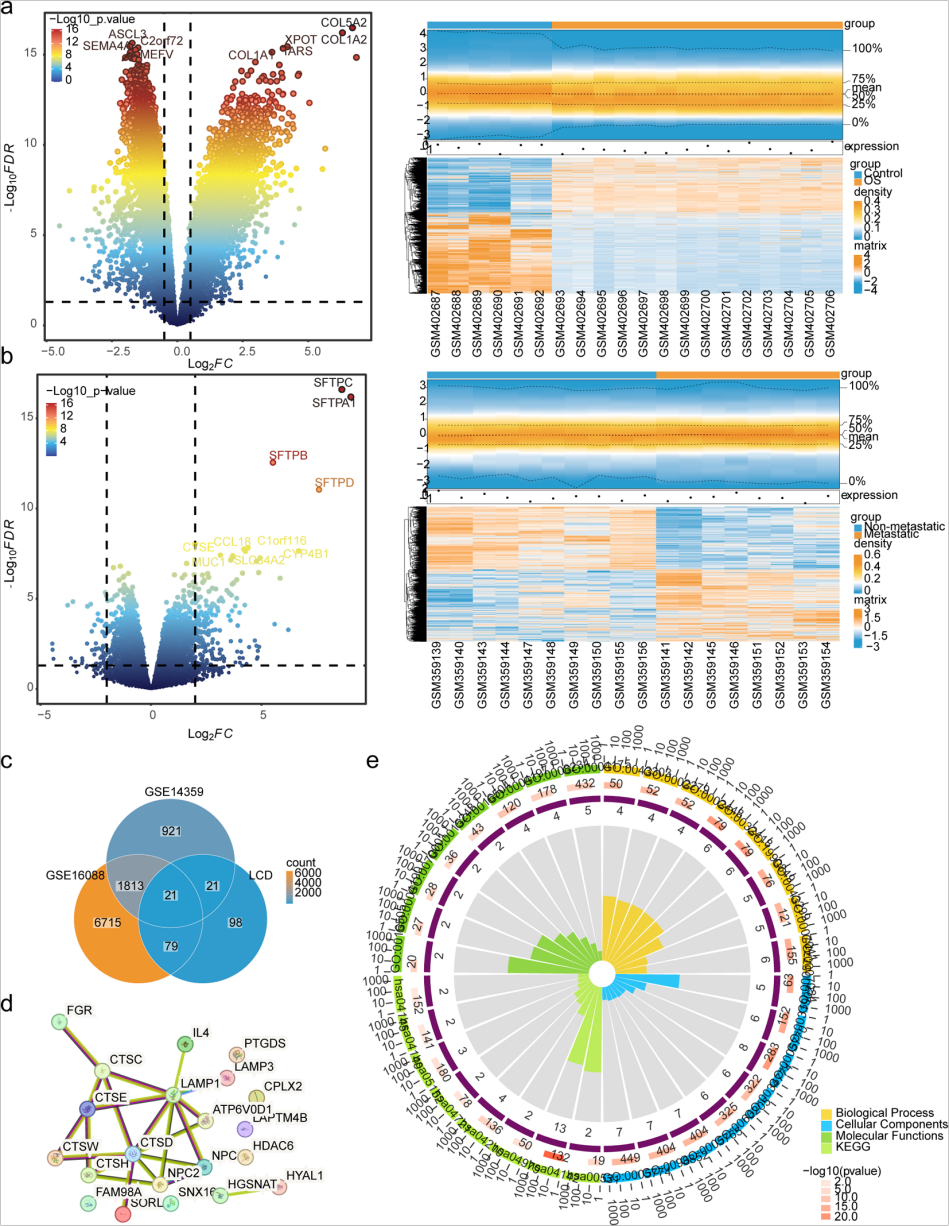
Identification and function analysis of DEGs. (**A**) Volcano plot (left) and heatmap (right) of DEGs1 between OS and control cohorts in GSE16088. (**B**) Volcano plot (left) and heatmap (right) of DEGs2 between metastasis and non-metastasis patients with OS in GSE14359. (**C**) Venn diagram displays the 21 intersection genes on overlapping DEGs1 from GSE16088, DEGs2 from GSE14359 and LCD-related genes. (**D**) The PPI network of 21 intersection genes. (**E**) Functional enrichment results for 21 intersection genes. DEGs, differentially expressed genes; OS, osteosarcoma; LCD, lysosomal-dependent cell death, PPI, protein-protein interaction

A total of 8,629 DEGs1 (3,686 upregulated and 4,943 downregulated) and 2,777 DEGs2 (1,484 upregulated and 1,293 downregulated) were identified in GSE16088 and GSE14359, respectively (Fig. 2A-B). By overlapping DEGs1, DEGs2 and LCD-RGs, 21 intersection genes were obtained (Fig. 2C), and the PPI network illustrated interactions among these 21 genes (Fig. 2D). For example, ATP6V0D1 interacted with LAMP1 and CTSD. Functional enrichment analysis revealed associations of intersection genes with 394 GO BP terms (e.g. ‘lysosome localization’, ‘lysosomal transport’, ‘vacuolar transport’), 63 GO CC terms (e.g. ‘lysosomal membrane’, ‘lytic vacuole membrane’, ‘vacuolar membrane’) and 64 GO MF terms (e.g. ‘sterol transporter activity’, ‘peptidase activator activity’, ‘endopeptidase activity’) (Fig. 2E). Additionally, eight functional pathways were enriched in KEGG, including ‘lysosomes’, ‘apoptosis’ and ‘autophagy’.

### Construction, evaluation and validation of the prognostic model

**Fig. 3:**
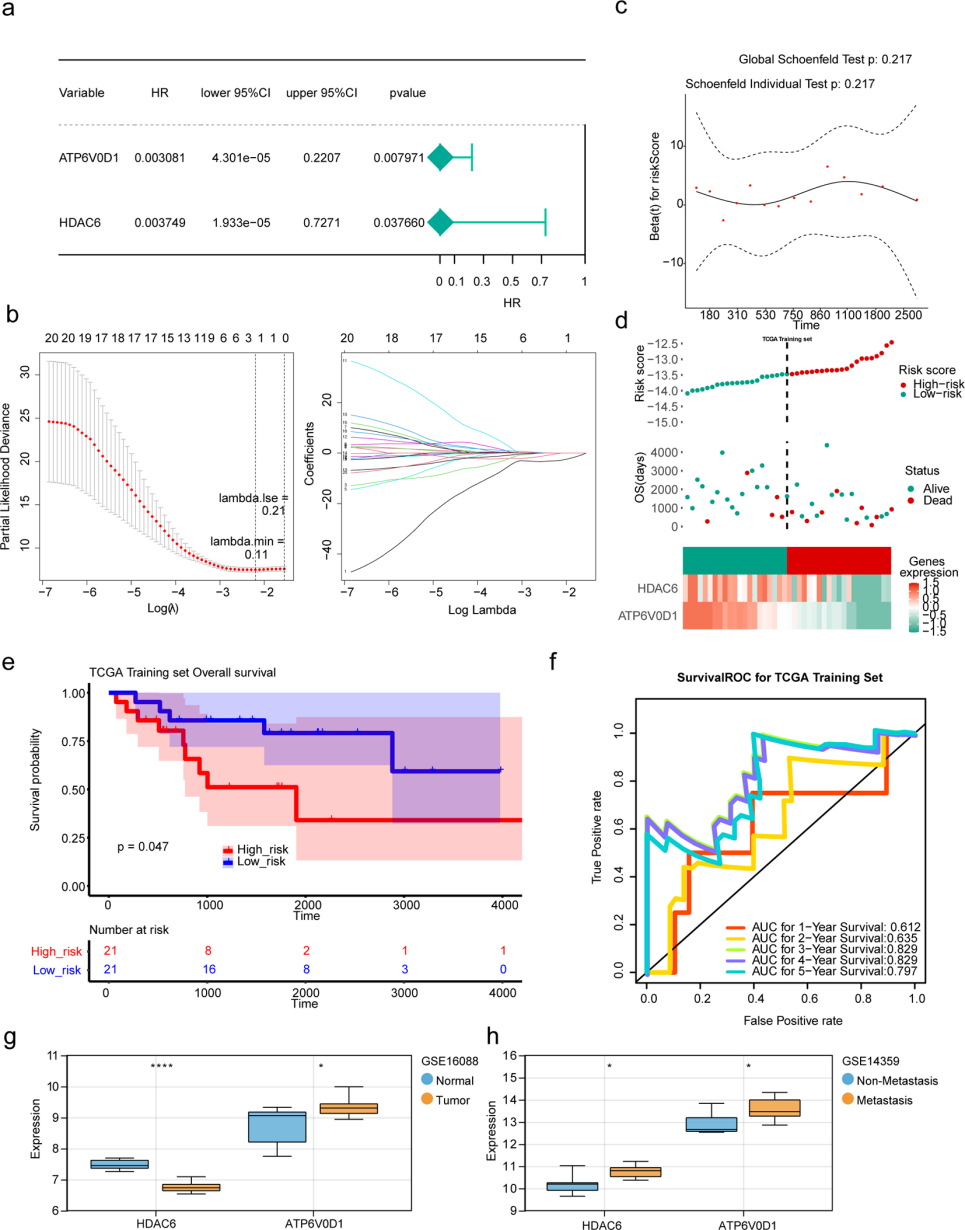
Construction of prognostic models. (**A**) The forest plot of univariate Cox analysis. (**B**) LASSO regression analysis for biomarkers. (**C**) PH assumption test. (**D**) Risk curve and heat map of gene expressions in high- and low-risk cohorts of the training set. (**E**) Kaplan–Meier curves for the high- and low-risk cohorts in the training set. (**F**) ROC curve in the training set. (**G**-**H**) The expression levels of ATP6V0D1 and HDAC6 in GSE16088 (**G**) and GSE14359 (**H**). LASSO, least absolute shrinkage and selection operator; PH, proportional hazards; ROC, receiver operating characteristic; HR, hazard ratio; lower 95%CI and upper 95%CI represent the 95% confidence intervals of the risk values

Two prognostic genes (ATP6V0D1 and HDAC6) were identified as biomarkers through univariate Cox and LASSO regression analyses based on the 21 intersection genes (Fig. 3A-B). The prognostic model passed the PH assumption test (*P* = 0.217), and risk scores were computed accordingly (Fig. 3C). Subsequently, risk curves and heatmap of gene expression in risk cohorts were generated based on risk scores (Fig. 2D). The risk score formula was determined as risk score = -4.24×ATP6V0D1+-2.20×HDAC6. K-M curves indicated a worse prognosis for patients with OS in the high-risk cohort (*P* = 0.047) (Fig. 3E), and the AUC values of ROC curves exceeded 0.6 at 1–5 years in the training set, indicating the valuable predictive performance of the prognostic model (Fig. 3F). Furthermore, expression levels of biomarkers revealed higher ATP6V0D1 expression in both metastatic and tumour samples, while HDAC6 expression was higher in normal as well as metastatic samples (Fig. 3G-H).

**Fig. 4:**
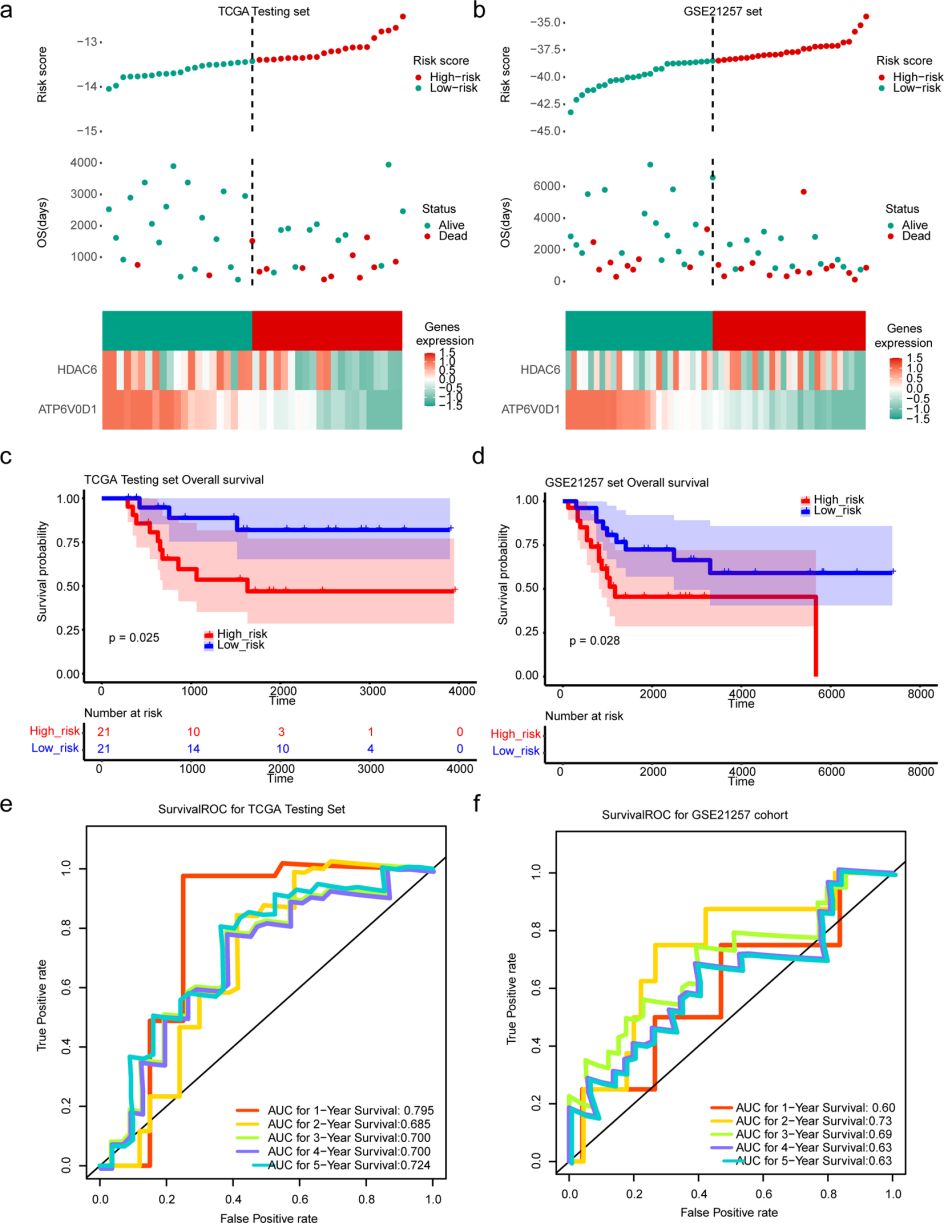
Validation of prognostic risk models. (**A**-**B**) Distribution of risk score and survival time, and heat map of biomarkers’ expression in the high- and low-risk cohorts in the testing (**A**) and GSE21257 (**B**) sets. (**C**-**D**) Kaplan–Meier curves in the testing (**C**) and GSE21257 (**D**) sets. (**E**-**F**) ROC curves in the testing (**E**) and GSE21257 (**F**) sets. ROC, receiver operating characteristic

Additionally, the prognostic model was validated using the testing set from TARGET-OS and GSE21257. Distribution of risk scores and survival time, as well as a heatmap of biomarker expression, were displayed for high- and low-risk cohorts in the testing set and GSE21257, respectively (Fig. 4A-B). K-M curves demonstrated higher survival probability for patients with OS in both the testing set and GSE21257, consistent with the training set (Fig. 4C-D). AUC values exceeding 0.6 at 1, 3 and 5 years in both the testing set and validation set indicated satisfactory predictive performance of the prognostic model (Fig. 4E-F).

### Distinct survival status and GSEA for risk cohorts

**Fig. 5:**
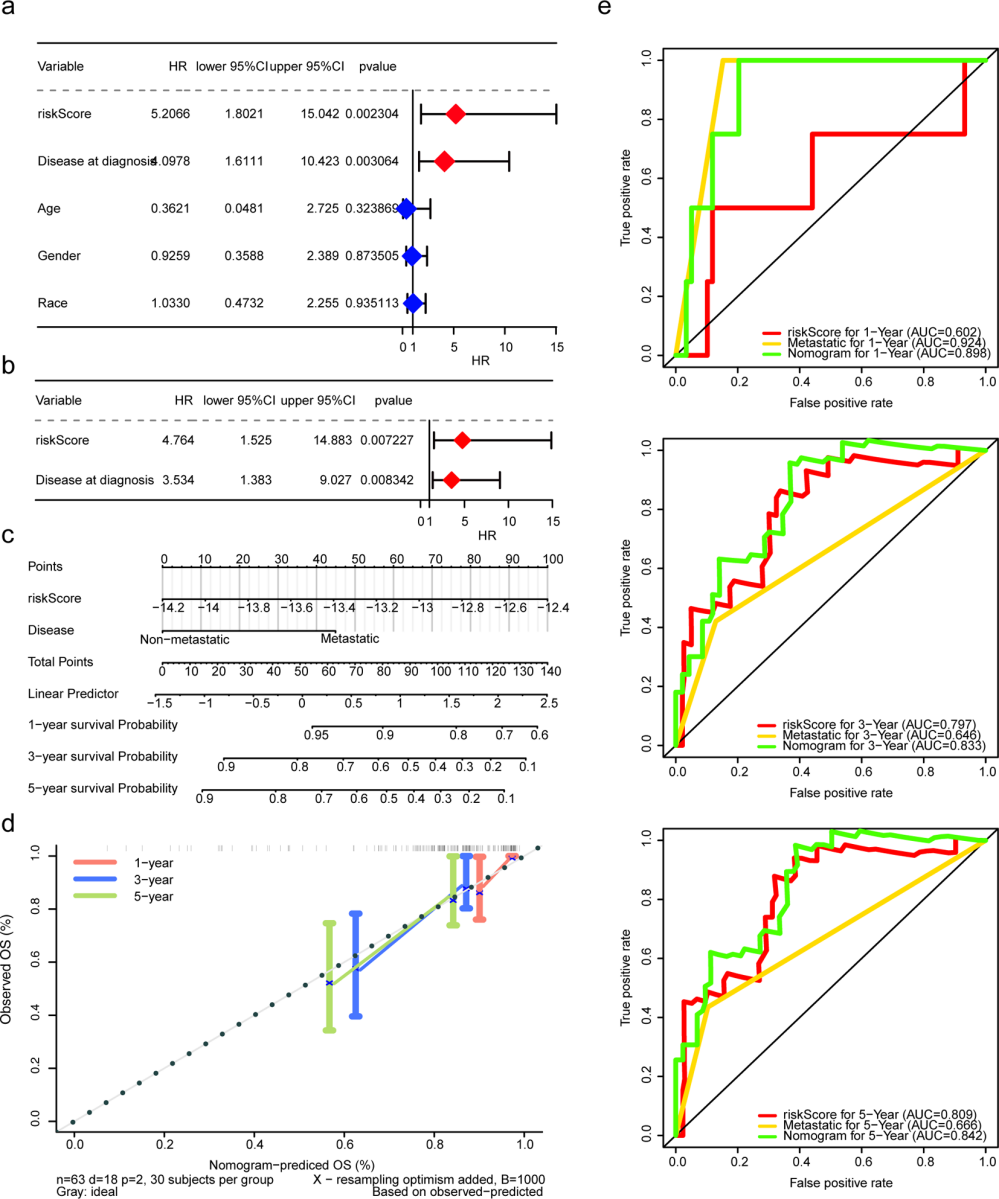
Construction and validation for the nomogram. (**A**) The forest plot of univariate Cox analysis. (**B**) The forest plot of multivariate Cox analysis. (**C**) A nomogram based on independent prognostic factors. (**D**) Calibration curves for the nomogram. (**E**) ROC curves: 1-year (top), 3-year (middle) and 5-year (bottom)

**Fig. 6:**
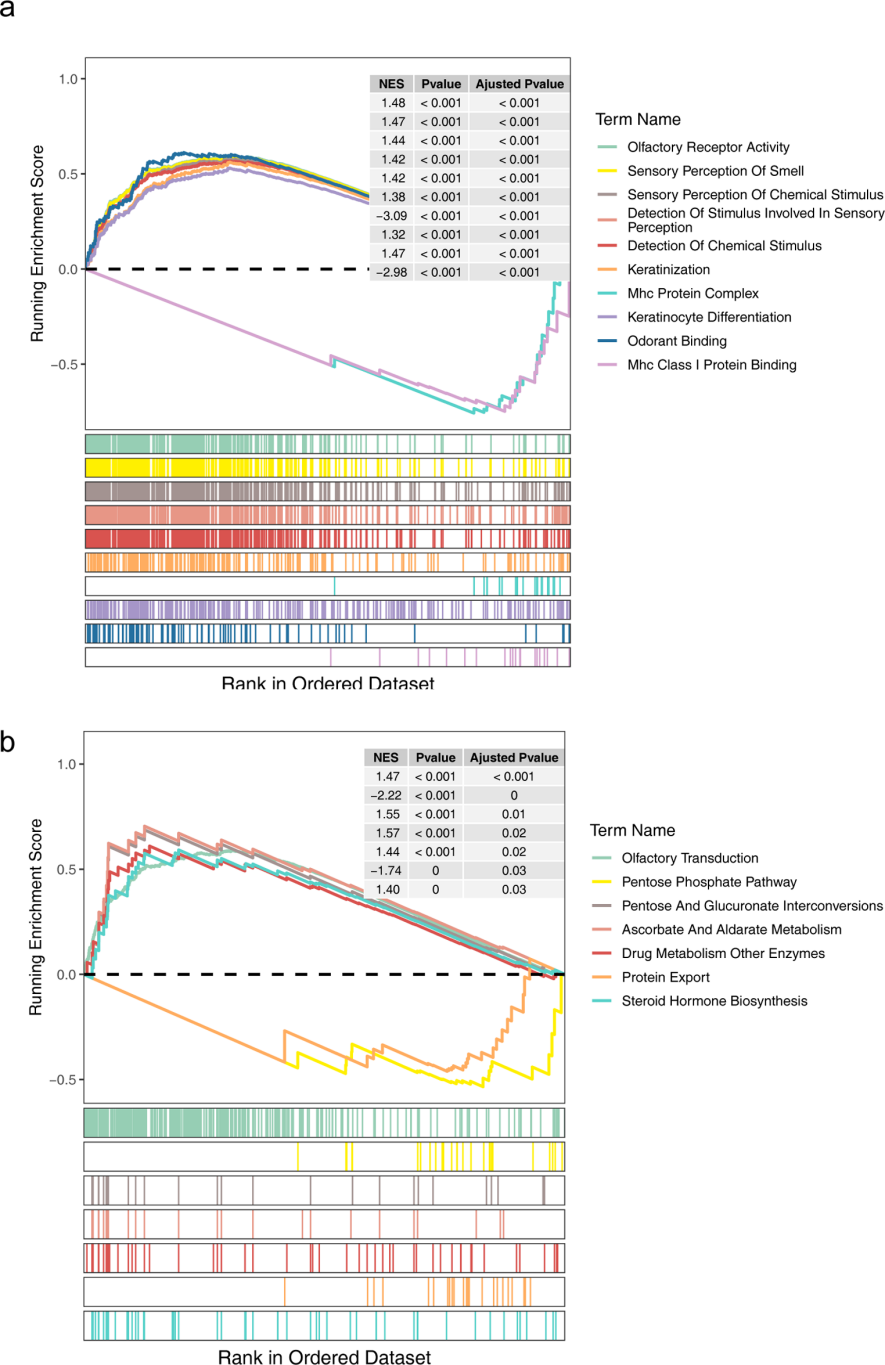
GSEA results for the high and low-risk cohorts. (**A**) GO enrichment profiles. (**B**) KEGG pathways. GSEA, gene set enrichment analysis; GO, Gene Ontology; KEGG, Kyoto Encyclopedia of Genes and Genomes

Significant differences in risk scores were observed in patient survival status for risk cohorts (Fig. S1A). Moreover, the distribution difference of patients from two risk cohorts in various clinical characteristics revealed higher mortality in the high-risk cohort compared to the low-risk cohort (Fig. S1B). Disease at diagnosis and risk score were identified as independent prognostic factors via Cox regression to construct a nomogram (Fig. 5A-C). Calibration and ROC curves suggested favourable prediction accuracy of the nomogram (Fig. 5D-E). GSEA conducted on both risk cohorts yielded 63 GO enrichment profiles and seven KEGG pathways (Fig. 6), indicating enrichment in various biological processes and pathways such ‘keratinocyte differentiation’, ‘odorant binding’ and ‘Mhc Class I protein binding’. Furthermore, GSEA of KEGG showed enrichment in ‘pentose phosphate pathway’ ‘protein export’ and ‘steroid hormone biosynthesis’.

### Immune microenvironment and anticancer drug prediction

**Fig. 7:**
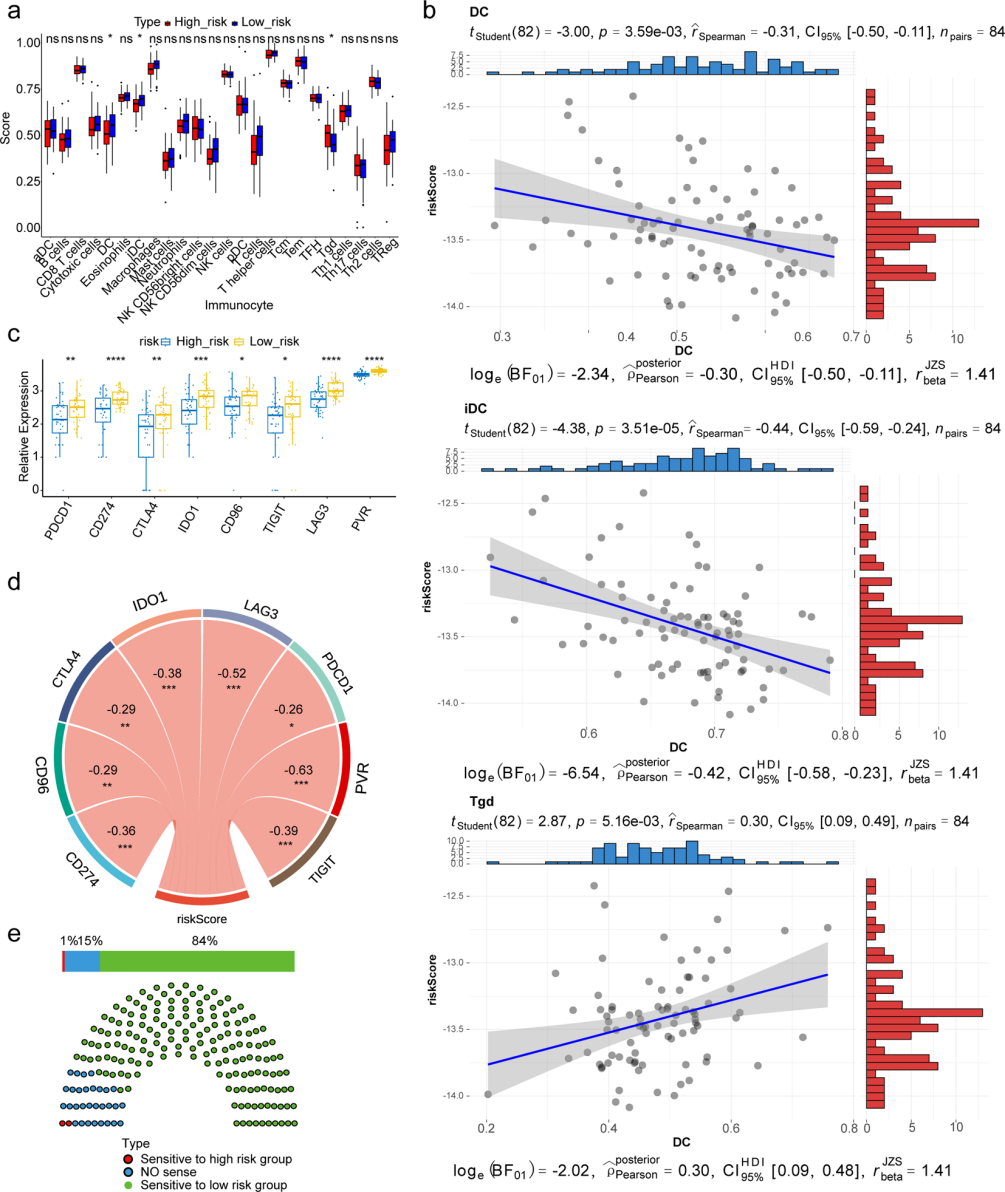
Immune microenvironment and drug sensitivity. (**A**) Box plot of immune cell percentage in high- and low-risk cohorts. (**B**) Scatter plot of correlation of risk scores and differential immune cells. (**C**) Relative expression of immune checkpoints in the high- and low-risk cohorts. (**D**) The correlation of risk score and differential immune checkpoints. (**E**) Drug sensitivity results for the high- and low-risk cohorts

Differential analysis of immune cells revealed statistically significant differences in dendritic cells (DCs), immature DCs (iDCs) and γδT cells (Tgd) between high and low-risk cohorts, suggesting potential roles of these immune cells in OS (Fig. 7A). Meanwhile, spearman correlation analysis indicated a significant association between risk scores and these three differential immune cells (Fig. 7B). Additionally, all immune checkpoints exhibited significant differences between the two cohorts, with CD274, IDO1, LAG3, PVR and TIGIT significantly correlated with risk score (|cor| > 0.3, Fig. 7C-D). Among the 198 anticancer drugs analysed, 168 showed significant differences between the risk cohorts, with the high-risk exhibiting greater sensitivity to two drugs (BI-2536_1086 and SB505124_1194), while the low-risk cohort showed greater sensitivity to 166 drugs (Fig. 7E).

### Importance of myeloid cells and osteoclasts in patients with OS

**Fig. 8:**
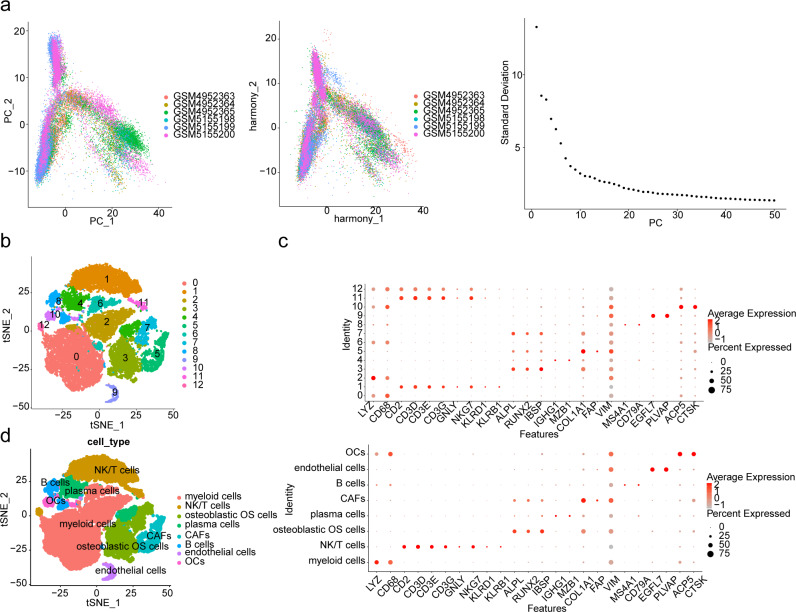
Acquisition of cell types. PCA results. (**A**) PCA of the dataset before correction (left), PCA of the dataset after batch correction (middle) and the standard deviation results (right). (**B**) High-quality cell clustering results. (**C**) Expression of marker genes in different cell clusters (up) and cell types (down). (**D**) High-quality cell annotation results. PCA, principal component analysis

**Fig. 9:**
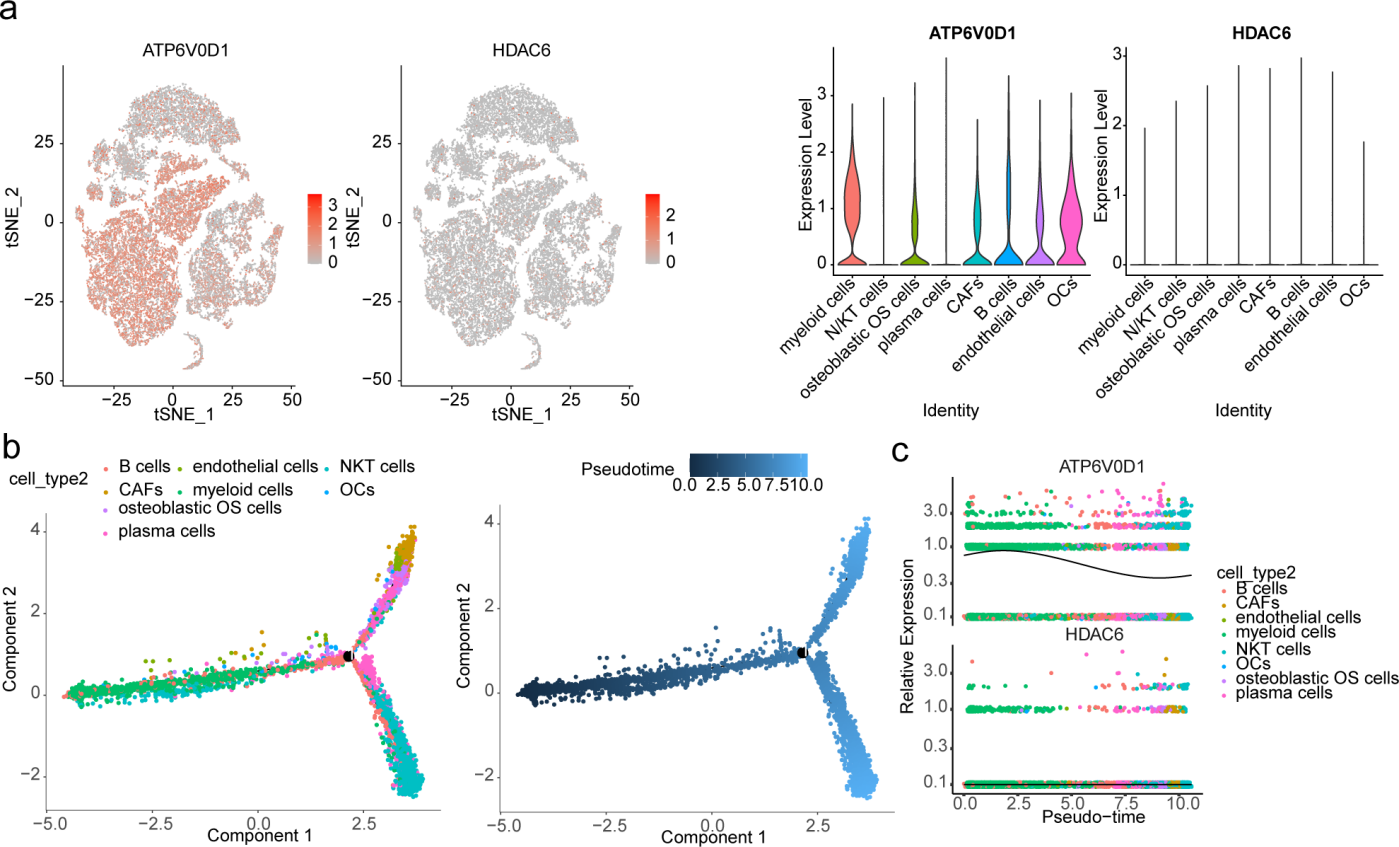
Pseudo-temporal analysis of biomarkers in single cells. (**A**) Biomarker expression in different cell types. (**B**) The single-cell trajectory map for eight cell types. (**C**) Pseudo-temporal trajectories for biomarkers

**Fig. 10:**
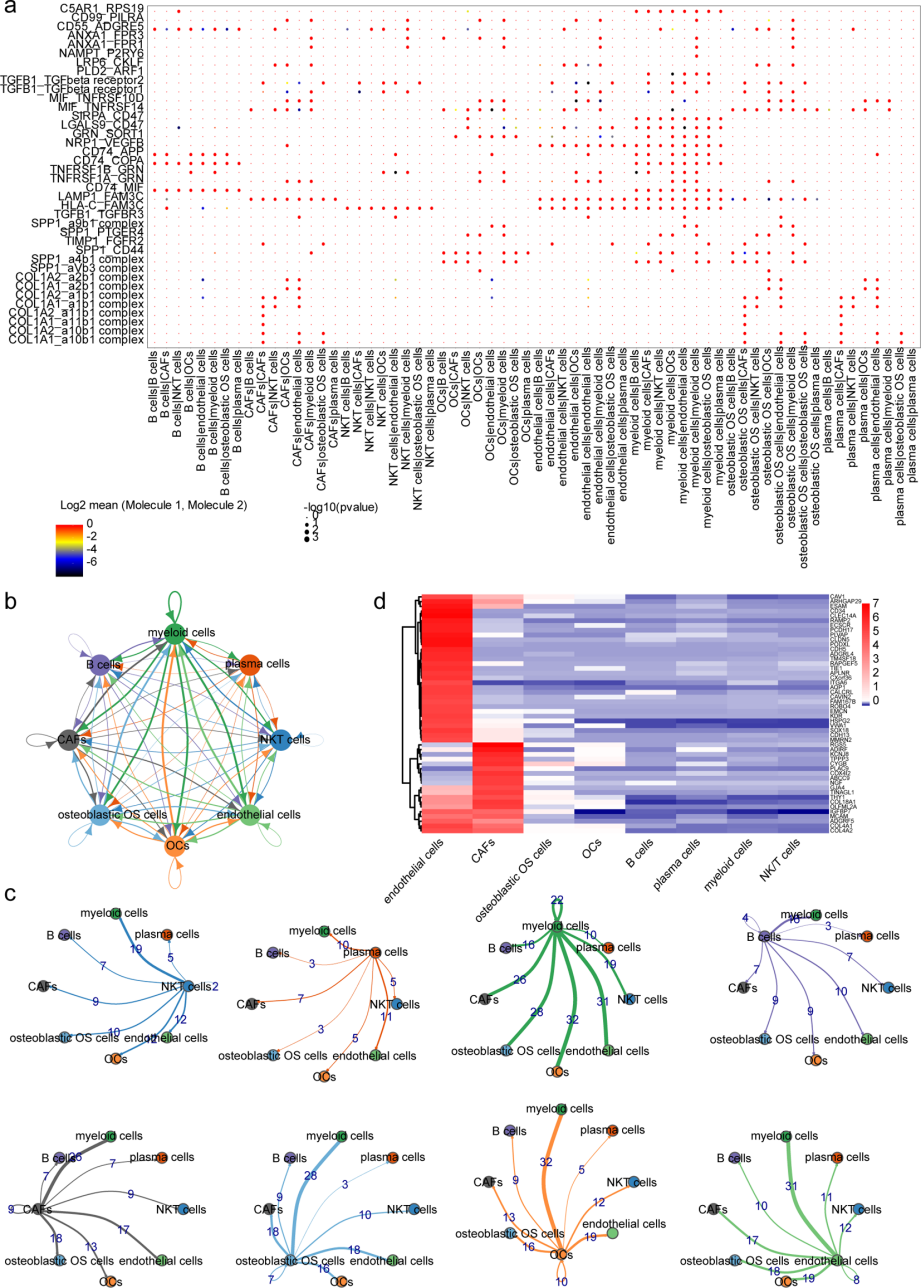
Analysis of cellular communication and transcription factors regulation in different cell types. (**A**) Scatter plot of ligand-receptor and multimeric interactions inter-cells. (**B**) Cell-cell interactions. (**C**) Cell-cell interactions in single cells. (**D**) The expression of transcription factors with significantly different activities in different cell types

An overview and filtration conditions of six samples from GSE162454 are presented in Table S1. Subsequently, 2000 highly variable genes were selected (Fig. S2), and PC30 was chosen for subsequent analysis (Fig. 8A). Cells were clustered into 13 classes using t-SNE (Fig. 8B) and annotated into eight types (myeloid cells, NK/T cells, osteoblastic OS cells, plasma cells, Cancer-associated fibroblasts (CAFs), B cells, endothelial cells, osteoclasts (OCs)) based on marker genes (Fig. 8C-D). Analysis of biomarker expression across different cell types revealed significant overexpression of ATP6V0D1 in myeloid cells and osteoclasts, while HDAC6 was under expressed across all cell types (Fig. 9A). Furthermore, the single-cell trajectory map indicated that myeloid cells and osteoclasts were the first cells to differentiate, suggesting their crucial importance in patients with OS (Fig. 9B). Notably, the expression of ATP6V0D1 progressively decreased over time (Fig. 9C). We identified a total of 38 ligand-receptor and multimeric interactions, such as LAMP1_FAM3C, between osteoblastic OS cells and OCs (Fig. 10A). Myeloid cells appeared to have a closer communication with other cell types, including osteoblastic OS cells, OCs and endothelial cells (Fig. 10B-C). Analysis of TF activity across different cell types revealed high expression of most TFs in endothelial cells and CAFs, suggesting potential influences on OS progression (Fig. 10D).

## Discussion

OS stands out as a highly lethal and metastatic malignant bone tumour. Tumor cell lysosomes, owing to their delicate nature compared to normal cell lysosomes, exhibit heightened susceptibility to LMP, potentially leading to LCD. While the correlation between LCD and tumour is established, there remains a dearth of research exploring the connection between LCD and OS. Thus, delving into the molecular mechanisms of LCD within OS holds promise for enhancing the prognostic outlook for patients with OS. In this study, we curated LCD-RGs from existing literature and identified DEGs through differential expression analysis. To further explore and enhance the predictive capacity of DEGs, we identified LCD-RGs (HDAC6 and ATP6V0D1) associated with OS as potential biomarkers. Leveraging LASSO and univariate Cox regression analyses, we constructed a prognostic model featuring an LCD-related gene signature.

Histone deacetylase 6 (HDAC6) has been implicated in various diseases, including neurological diseases [24], heart diseases [25] and inflammatory diseases [26]. Moreover, emerging evidence underscores its involvement in tumorigenesis and metastasis. For instance, Ying et al. demonstrated that downregulation of HDAC6 suppressed proliferation, migration, invasion and apoptosis resistance in HPV-positive cervical cancer cells, inhibiting the growth and metastasis of xenograft tumours in vivo [27]. HDAC6 has exhibited oncogenic properties across several cancer types, including endometrial cancer [28], breast cancer [29], and esophageal cancer [30]. While research on the association between HDAC6 and OS remains limited, discussions on HDAC6 inhibitors for OS treatment hint at a potential link, offering a novel therapeutic avenue. For instance, Jun et al. demonstrated that WT161 (an HDAC6 inhibitor) inhibited OS cell growth and enhanced apoptosis, synergizing with 5-FU in killing OS cells in vitro and in vivo [31]. However, robust large-scale studies are imperative to establish a direct correlation between HDAC6 expression and clinical outcomes in patients with OS. Our findings indicate an association between HDAC6 expression and LCD in terms of the prognosis of patients with OS. Furthermore, Xu et al. provided evidence suggesting HDAC6’s pivotal role in cancer immunomodulation. Their study showcased heightened M2 macrophage infiltration in HDAC6-overexpressing colon cancer tissues, elucidating the HDAC6-TAK1-ADAM17 regulatory axis in sil-6R release and macrophage polarization in colon cancer [32]. Further investigation is warranted to delineate the pathway through which HDAC6 impacts OS progression via LCD and modulates immunological processes.

As encoding a protein crucial in forming vacuolar ATPase (V-ATPase) [33], V-type proton ATPase subunit d1 (ATP6V0D1) has been implicated in various cancer hallmarks, particularly invasion and metastasis. Inhibition of V-ATPase emerges as a potential anti-cancer therapeutic strategy [34]. V-ATPases are closely associated with autophagy [35, 36] and the endocytic uptake of extracellular fluid, which are significant pathways supplying nutrients to cancers [37]. The overexpression of V-ATPases correlated positively with invasion and metastasis. For example, Sennoune et al. demonstrated greater V-ATPase activity in highly metastatic breast cancer cells compared to lowly metastatic ones, with V-ATPase inhibitors reducing invasion and migration in highly metastatic cells [38]. In this research, we revealed elevated ATP6V0D1 expression in both metastatic and tumour samples compared to normal ones. Besides, the result shown in the heatmap of ATP6VOD1 expression in the high-and low-risk cohorts aligns with recent findings, which have demonstrated that ATP6V0D1 serves as a protective for OS [39, 40]. Therefore, the ATP6V0D1 may react actively as a protective factor inhibiting tumour growth and progression through disruption of pH homeostasis, suggesting a possible defending mechanism against tumour. Chen et al. found that ATP6V0D1-mediated inhibition of the signal transducer and activator of transcription 3 (STAT3) increases alkaliptosis in pancreatic ductal adenocarcinoma cells and a high expression of ATP6V0D1 correlates with prolonged survival of patients with this carcinoma [41]. The induction of alkaliptosis, a type of regulated cell death driven by intracellular alkalization, plays an important role in tumor immunity and inflammatory response [42]. Meanwhile, it has been proved that caspase-8 (a well characterized initiator of apoptosis) can block the assembly of functional V-ATPase through binding to the V0 domain of V-ATPase, but not the V1domain, which leads to lysosomes alkalinization and, eventually, may induce LMP [43]. However, while ATP6V0D1 serves as a prognostic biomarker for OS, its direct impact on OS progression remains uncertain, necessitating further exploration. Furthermore, to further understand the contextual roles of different V-ATPase isoforms will in tumour biology is of benefit in precision medicine research.

Further analysis of clinical characteristics identified metastatic status as a pivotal variable influencing outcomes in patients with OS. To enhance survival prediction, we developed a nomogram integrating risk score with disease at diagnosis. The performance of this nomogram was assessed through time ROC curves at 1, 3 and 5 years, along with calibration plots, demonstrating a satisfactory fit of predicted and observed outcomes, affirming the predictive capability of our model.

The tumour microenvironment (TME), comprising cellular and acellular components, can reprogram tumour initiation, growth, invasion, metastasis and response to therapy [44]. Immune infiltration within the tumour microenvironment plays a crucial role in cancer progression and clinical prognosis [45]. Thus, understanding the tumour environment is essential for identifying immune modifiers of cancer progression and developing immunotherapies, offering insights into OS treatment strategies [46]. In our study, utilising the ssGSEA algorithm and Wilcoxon test, we elucidated the composition of immune cells within the OS TME across two cohorts. Additionally, we found that higher levels of Tgds, alongside lower levels of DCs and iDCs, correlated with the high-risk cohort exhibiting poorer prognosis among patients with OS, indicating an association between LCD-related risk and immune characteristics.

DCs are a family of professional antigen-presenting cells, which play a crucial role in initiating innate and adaptive immune responses against pathogens and tumour cells [47]. Muraro et al. reported a lower phenotype expression of maturation markers of the DCs, which were co-cultured with the OS cell lines, implying that OS highly interferes with an in vitro DCs immune function as antigen-presenting cells [48]. Generally, mature DCs have been traditionally associated with immune stimulation, while iDCs have been associated with immunosuppression and tolerogenicity. However, evidence indicates that the specific subset type and maturation status of DCs influence the characteristics of immune responses and subsequently impact cancer prognosis [49, 50]. Similarly, Tgds, vital components of tumour effector cells, exert dual roles in tumour immunity. are divided into two primary subsets in humans based on their T cell receptors: Vδ1 T cells and Vδ2 T cells [51]. Moreover, Tgds contribute to immunosurveillance against tumours, For instance, Cordova et al. demonstrated that Tgds represent the major lymphocyte population infiltrating melanoma, where both Vδ1 T cells and the Vδ2 T cells are involved, and kill melanoma cells [52]. Additionally, Aggarwal et al. concluded that Tgds enhance immune surveillance via macrophage infiltration and improve antigen presentation [53]. However, tumour infiltrating Tgds have also been demonstrated to promote tumor development and metastasis [54]. For instance, Ma et al. observed significantly increased Tgd expression in breast tumor tissues (43 of 46 tumor samples) compared to normal breast tissues (2 of 46 normal samples), wherein the number of tumor-infiltrating Tgds positively correlated with advanced tumor stages, but inversely with the overall survival of patients with breast cancer [55]. Overall, unrevealing the complex interplay between immune cells and their subsets within the OS tumour microenvironment is crucial for developing effective therapeutic strategies and improving patient outcomes. Further investigation into the underlying mechanisms governing these interactions is warranted to advance our understanding of OS pathogenesis and treatment.

Tumor cells often activate immune checkpoint pathways to suppress antitumor immune responses, evading immunosurveillance and promoting progression [56]. The advent of immune checkpoint inhibitors has revolutionised treatment approaches for various malignancies [57], presenting a promising avenue for the therapeutic management of OS, which has seen little progress in decades. Currently, mono- or dual-therapy with checkpoint inhibitors in OS has failed to yield significant anti-tumour efficacy and, on the other hand, most patients treated with immune checkpoint inhibitors exhibit limited responses [58]. In our study, we observed significantly higher expression levels of CD274, ID01, LAG3, PVR and TIGIT in the low-risk cohort, suggesting that patients in this category may exhibit enhanced immunoreactivity to immune checkpoint inhibitors. Clarifying the availability and validity of predictive biomarkers, along with identifying new prognostic biomarkers through clinical studies of immune checkpoint inhibitors in OS, holds the potential for informing treatment paradigms.

Furthermore, we identified differential drug sensitivities based on the risk categorization associated with the two LCD-RGs. The high-risk cohort demonstrated greater sensitivity to two drugs (BI-2536_1086 and SB505124_1194), while the low-risk cohort exhibited enhanced sensitivity to 166 drugs (Fig. 9E), providing potential drug alternatives tailored to each risk cohort.

Finally, to understand the functions and interactions of the prognostic-related LCD-RGs at the cellular level, we categorised cells into eight types through cluster analysis and annotation combined with marker genes. These comprised four non-immune cells (i.e., osteoblastic OS cells, plasma cells, CAFs, endothelial cells) and four immune cells. Our findings revealed a progressive decrease in ATP6V0D1 expression over time, suggesting a greater role for ATPV0D1 in myeloid cells and osteoclasts, as evidenced by pseudotime analysis. This perspective offers novel insights for identifying precision therapeutic targets.

## Conclusions

This study introduces and validates a unique prognostic model based on the LCD-RGs (ATP6V0D1 and HDAC6) for OS. We demonstrate a significant association between risk categorization based on LDC-RGs with patient survival, underscoring the clinical relevance of our model. Furthermore, we delve into the complex association between immune cell infiltration in the TME and differentiated risk cohorts based on LCD-RGs, providing valuable insights for tailoring immunotherapeutic strategies. Additionally, our analysis of drug sensitivities within different risk cohorts offers promising avenues for precision medicine. Moreover, through analysis at the cellular level, we revealed the interactions of LCD-RGs with cells of the TME, offering fresh perspectives on precision medicine approaches in OS treatment. However, this study has several limitations: (1) Our analytical approach primarily relies on bioinformatics, necessitating biological experiments in subsequent research to validate our findings. (2) The limited sample size of the dataset we utilised underscores the need for expanded research scopes in follow-up studies to consolidate evidence and enhance the predictive reliability of our model. (3) Lastly, elucidating the exact mechanisms through which LCD-RGs modulate immune cells in the TME and drug sensitivity necessitates further extensive research. Therefore, a comprehensive investigation to unravel the intricate interactions between LCD-RGs and OS pathogenesis is essential.

### Electronic supplementary material

Below is the link to the electronic supplementary material.

Supplementary Material 1

## Data Availability

All data generated or analyzed in this study are included in this article. The datasets generated and/or analyzed in this study are available online (The Cancer Genome Atlas (TCGA, https://portal.gdc.cancer.gov/) and Gene Expression Omnibus (GEO, https://www.ncbi.nlm.nih.gov/geo/).

## Abbreviations

OS: Osteosarcoma
LCD: lysosomal-dependent cell death
LMP: lysosomal membrane permeabilization
LCD-RGs: lysosomal-dependent cell death-related genes
TCGA: The Cancer Genome Atlas
GSE: gene expression omnibus series
GEO: Gene Expression Omnibus
DEGs: Differentially expressed genes
PPI: protein-protein interaction
GO: Gene Ontology
CC: Cellular Components
MF: Molecular Functions
BP: Biological Process
KEGG: Kyoto Encyclopedia of Genes and Genomes
LASSO: least absolute shrinkage and selection operator
K-M: Kaplan-Meier
ROC: receiver operating characteristic
AUC: area under the curves
GSEA: gene set enrichment analysis
ssGSEA: single sample gene set enrichment analysis
IC50: half maximal inhibitory concentration
GDSC: Genomics of Drug Sensitivity in Cancer
PCA: principal component analysis
PCs: principal components
TF: transcription factor
DC: dendritic cells
iDCs: immature DCs
Tgd: γδT cells
CAFs: cancer-associated fibroblasts
OCs: osteoclasts
HDAC6: histone deacetylase 6
ATP6V0D1: V-type proton ATPase subunit d1
V-ATPase: vacuolar ATPase
STAT3: signal transducer and activator of transcription 3
TME: Tumour microenvironment
