# Supplementary material for: Integrated analysis of single-cell and bulk RNA sequencing data reveals prognostic characteristics of lysosome-dependent cell death-related genes in osteosarcoma
Source: BMC Genomics. 2024 Apr 17;25:379. doi: 10.1186/s12864-024-10283-5 (PMC11022332; doi:10.1186/s12864-024-10283-5)
Supplement: Supplementary file 1 — Supplementary Material 1 [file 12864_2024_10283_MOESM1_ESM.pdf]

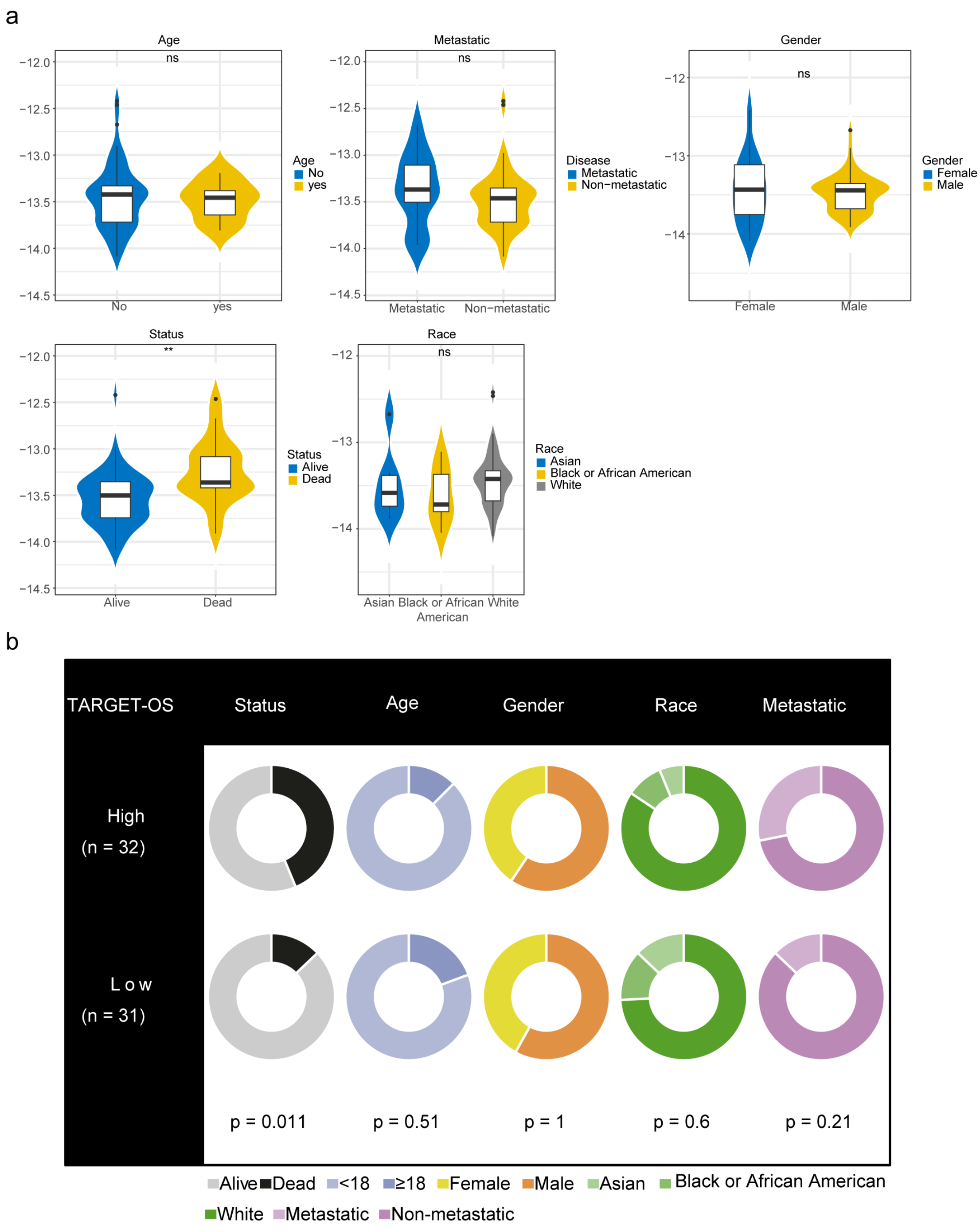

Figure S1. Clinical characteristics analysis. (A) Comparison between subgroups with different clinical characteristics (age, metastatic, gender, status and race). (B) Distribution of risk scores among patients with the five clinical characteristics. *P < 0.05, **P < 0.01, ***P < 0.001, ****P < 0.001, ns: non-significance.

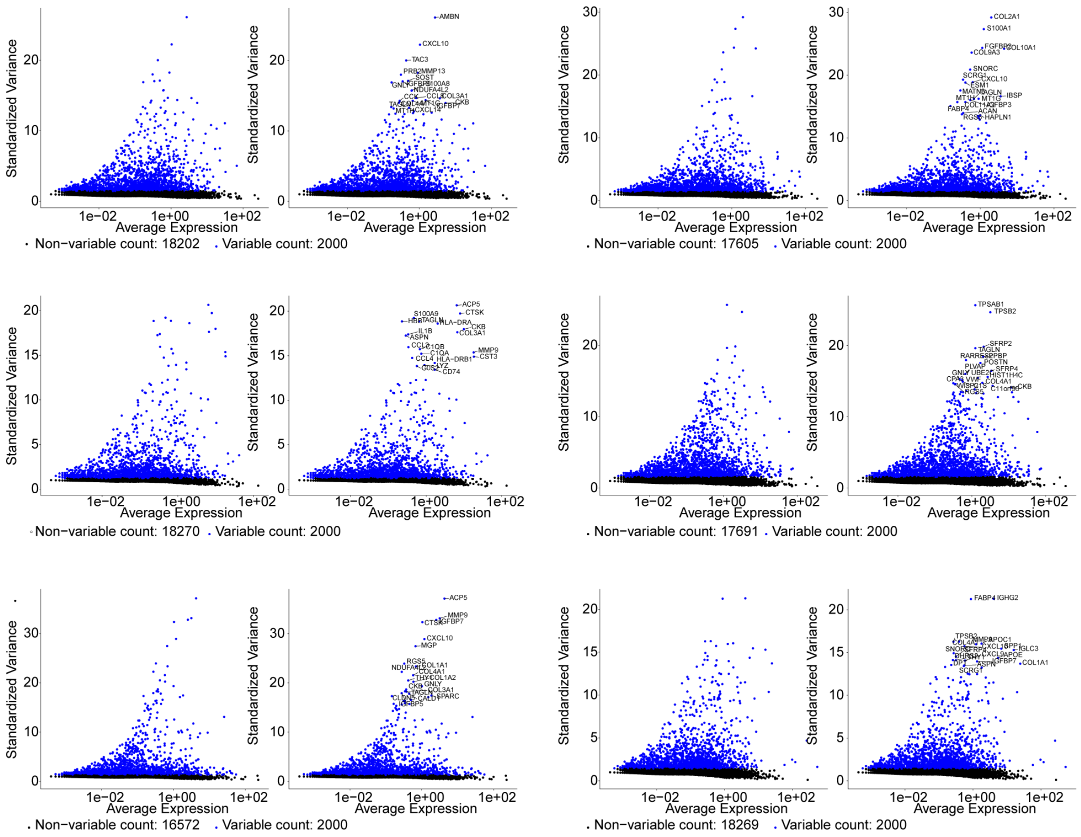

Figure S2. The 2000 highly variable genes in six single-cell samples.

Table S1 Cellular filtration of the six samples

Sample	Rawdata	ByLibSize	ByFeature	ByMT	ByDoubletScore	Remaining	percent
GSM4952363	9345	532	532	880	1047	6354	0.680
GSM4952364	8841	500	499	1023	814	6005	0.679
GSM4952365	10630	593	593	1778	956	6710	0.631
GSM5155198	4479	134	134	321	343	3547	0.792
GSM5155199	11568	794	794	1024	1300	7656	0.662
GSM5155200	10136	575	574	756	1215	7016	0.692

Table S1 Cellular filtration of the six samples.
